# Erectile dysfunction and associated risk factors in male patients with ischemic stroke: A cross-sectional study

**DOI:** 10.1097/MD.0000000000018583

**Published:** 2020-01-03

**Authors:** Hengheng Dai, Jisheng Wang, Qi Zhao, Jianxiong Ma, Xihao Gong, Lu Wang, Binghao Bao, Haisong Li, Bin Wang

**Affiliations:** a Department of Andrology, Dongzhimen Hospital, Beijing; b Department of Andrology, Hang Zhou Red Cross Hospital, Hangzhou, China.

**Keywords:** cross-sectional study, erectile dysfunction, ischemic stroke, risk factor

## Abstract

Patients with ischemic stroke (IS) often suffered from the problem of erectile dysfunction (ED) and psychological disease. However, they are often ignored because these symptoms are more obvious in the convalescent stage of stroke, which affects the quality of sexual life of patients. This study aimed to investigate the incidence of ED, sexual quality of life, and mental state of patients after stroke, as well as analyze the relevant risk factors affecting their psychological status.

A total of 361 IS patients were enrolled. The international erectile function index-5 scale was used to diagnose ED. Accordingly, the patients were divided into ED group and non-ED group. Magnetic resonance imaging was used to evaluate the brain lesions of patients. We assessed neurological deficits by the National Institutes of Health Stroke Scale score and patient health questionnaire-9 (PHQ-9) and general anxiety disorder-7 (GAD-7) were used to evaluate the depression and anxiety. The differences between the ED group and the non-ED group clinical factors were compared.

The response rate was 88.6% (n = 320), and more than two-thirds of patients reported ED (77.8%). Patients with ED had higher PHQ-9 (8.40 ± 4.18 vs 4.94 ± 3.73, *P* < .01) and GAD-7 (6.73 ± 3.56 vs 4.51 ± 3.35, *P* < .01) scores, were more likely to have the frontal lobe (75.1% vs 49.3%, *P* < .01) and lateral ventricle (69.8% vs 53.5%, *P* = .01) lesions, with hypertension (75.1% vs 46.5%, *P* < .01) and hyperlipidemia (48.2% vs 25.4%), and on antihypertensive (67.9% vs 35.25, *P* < .01) and hypolipidemic drug (43.4% vs 16.9%, *P* < .01). Multivariate logistic regression analysis showed that antihypertensive drug (odds ratio [OR]: 2.50, 95% confidence interval [CI]: 1.02–6.10, *P* = .04), depression (OR: 1.18, 95% CI: 1.06–1.32, *P* < .01) and anxiety (OR: 1.13, 95% CI: 1.01–1.27, *P* = .04) might be the independent risk factors for ED group.

ED is more common in male IS patients. Antihypertensive drug, depression and anxiety are the main factors affecting ED.

## 1 Introduction

Ischemic stroke (IS) refers to the sudden decrease or stop of local blood supply to the brain tissue, resulting in ischemia and hypoxia, leading to necrosis and softening of the brain tissue, accompanied by clinical symptoms such as hemiplegia and aphasia.^[1]^ At present, IS is one of the leading cause of death and disability in the world. The number of deaths due to IS in China is second only to tumors. Epidemiological studies have shown that there are between 1.5 million and 2 million new IS in China each year.^[2,3]^

Erectile dysfunction (ED) is manifested by the penis unable to reach or maintain sufficient hardness to obtain a more satisfactory coitus.^[4,5]^ ED can be caused by many different diseases, including psychological stress, vascular disease, decreased hormone levels, relationship between husband and wife, and socioeconomic status.^[6,7]^ In addition to causing limb movement, psychological and cognitive impairment, IS also has important impacts on the patient's sexual function, especially sexual life frequency, erectile function, sexual desire, sexual excitement, and so on. However, there is limited information on the incidence of ED in men and related risk factors. Whether IS is an explicit cause for ED is uncertain. Therefore, this study aimed to investigate the erectile function of male patients after IS, and analyze the influencing factors related to erectile function.

## 2 Method

This study was a cross-sectional and clinical-based survey and had been approved by the Ethics Committee of Dongzhimen Hospital Affiliated to Beijing University of Chinese Medicine. Male stroke patients who hospitalized in the Department of Neurology, Dongzhimen Hospital from June 2016 to January 2018 were investigated by questionnaire. The inclusion criteria were:

(1) male patients aged ≤65 years;
(2) met the diagnostic criteria for IS^[8]^;
(3) stable for at least 6 months;
(4) those who volunteered to complete the questionnaire.

Exclusion criteria were:

(1) sexual dysfunction before the patient's stroke;
(2) patients with unconsciousness or severe language dysfunction after the onset of illness, inability to make informed consent or adequate cooperation in the study;
(3) local lesions that affect erectile function before onset, such as penile sclerosis, anatomical malformation, penile cancer, or neurosis;
(4) pre-onset severe arrhythmia, severe liver and kidney dysfunction, severe lumbar disc herniation, hematopoietic system disease, patients with neurological diseases;
(5) history of pelvic surgery before onset.

During the visit survey, these subjects completed the questionnaire independently to the doctor and/or medical visitor. Before participating, each participant was informed of the research purpose and privacy, and participation in the study was entirely voluntary and anonymous.

**Figure 1:**
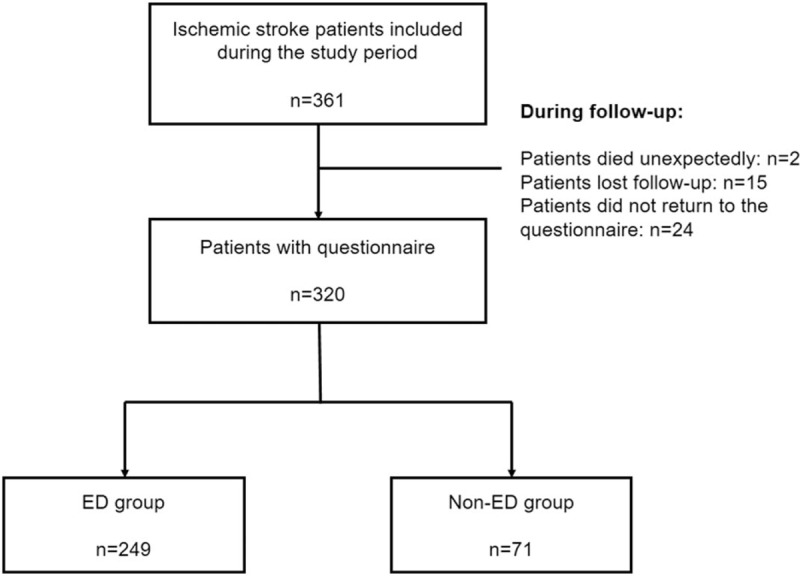
Flow chart of the study population.

In this survey, each enrolled patient would be followed up 6 months after IS to investigate the patient's erectile function and related risk factors. A total of 361 subjects were eligible for inclusion and exclusion. Among them, 2 patients died unexpectedly during follow-up, 15 patients lost follow-up, and 24 patients did not return to the questionnaire. Finally, 320 patients completed the survey, with a total response rate of 94.40% (Fig. 1).

The severity of stroke-induced neurological damage was assessed by the National Institutes of Health Stroke Scale (NIHSS). The NIHSS score ranged from 0 to 42 and the higher the score was, the more severe the neurological damaged.^[9]^ Magnetic resonance imaging (MRI) data were collected from all admitted patients. The simple depression and anxiety rating scale patient health questionnaire-9 score and general anxiety disorder-7 score scale were used to evaluate patients’ anxiety and depression status.^[10–12]^ The international index of erectile function-5 (IIEF-5) scale was used to diagnose the ED in patients 6 months after stroke.^[13]^ The subjects were divided into ED group (<22 points) and non-ED group (≥22 points) according to the score. A set of identified risk factors for stroke or factors were record, including hypertension (using antihypertensive drugs or systolic blood pressure >140 mm Hg or diastolic blood pressure >90 mm Hg), diabetes (using insulin or oral antidiabetic treatment). Two glucose during hospitalization (>7 mmol), hypercholesterolemia (using lipid-lowering drugs or low-density lipoprotein cholesterol >1 g /L), current smoking, drinking and overweight, (body mass index (BMI) ≥26 kg/m^2^).

All data were analyzed and processed by SPSS 21.0 as statistical software. The measurement data were expressed as mean ± standard deviation 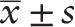, and *t* test or nonparametric test was used for comparison between groups. The 2 sample rates were compared between the count data sets using the *χ*^2^ test. Logistic regression analysis was performed on risk factors associated with the disease. *P* < .05 had a statistically significant difference.

## 3 Result

Of the 320 patients enrolled in the study, the mean age was 55.58 ± 6.3 years, and 88.8% and 74.4% of patients reported having the habit of smoking and drinking. The mean BMI was 26.11 ± 2.63, which was in the overweight range. A total of 249 patients had ED, the incidence rate was 77.8%. There was no significant difference in the age, smoking, alcohol consumption and BMI between the ED group and the non-ED group (*P* > .05).

Compared with the non-ED group, the IIEF-5 score was significantly lower in the ED group. The decrease in the NIHSS score also showed that the ED group had a more severe stroke. Correspondingly, anxiety and depression were also more severe in the ED group. According to the MRI, the infarction of the cerebral infarction caused by stroke patients was mainly, and the infarction in a single site was rare. There were 257 cases of multi-site infarction, and 63 cases of single infarction. The location of the cerebral infarction was concentrated in the frontal lobe, lateral ventricle, parietal lobe, basal ganglia, and thalamus. Compared with the non-ED group, 75.1% of patients had infarction in the frontal lobe, 73.9% had lesions in the lateral ventricle (*P* = .01), and 18.1% in the temporal lobe.

The incidence of hypertension and hyperlipidemia was significantly higher in patients with non-ED group, but there was no significant difference in the incidence of coronary heart disease and diabetes. Correspondingly, in the past, the use of antihypertensive drugs and statins in the ED group was significantly higher than that in non-ED patients. There was no significant difference in the use of hypoglycemic agents in patients with ED compared with non-ED patients (Table 1).

**Table 1:**
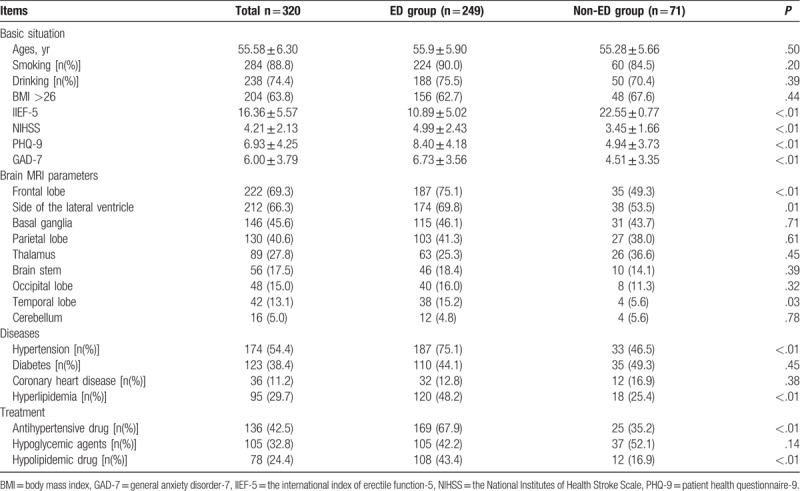
Clinical and radiological characteristics of IS patients with and without ED.

Univariate analysis of ED-related factors reveals 6 suspicious factors associated with hypertension, hyperlipidemia, antihypertensive drug, hypolipidemic drug, anxiety, and depression. Multivariate logistic regression analysis shows that antihypertensive drug, depression and anxiety may be the independent risk factors for ED group (Table 2).

**Table 2:**
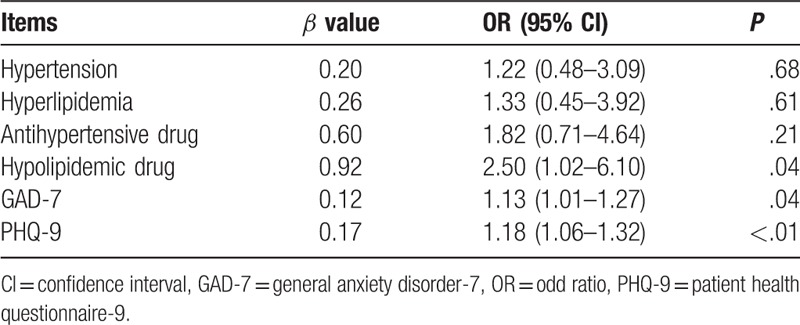
Multivariate Logistic regression analysis of erectile dysfunction associated risk factors.

## 4 Discussion

This study found that the occurrence of ED after IS was 77.8%, significantly higher than the common ED. According to a recent survey in China, the incidence of ED is only 40.56% among men over 40 years old.^[14]^ For the high prevalence of ED after IS, there are some possible explanations:

(1) the advanced central nervous system that regulates sexual function is located in the cerebral cortex and subcortical center, mainly sensing visual, auditory, tactile, olfactory, and hallucinatory to induce sexual impulsivity.^[15]^ After stroke, patients’ brain function impaired and nerve sensitivity decreased, leading to the appearance of ED.
(2) The patients surveyed in this study were older and often accompanied by a variety of underlying diseases, which could affect the erectile function. Sexual dysfunction in patients after IS mainly includes sexual decline in sexual frequency,^[16]^ decreased sexual satisfaction,^[17]^ and emotional changes.^[18]^

A case-control study of up to 7 years of follow-up showed that the life satisfaction of the spouse of the stroke survivor was significantly lower than that of the control group. The life satisfaction of the spouse is related to the survivor's age, gender, support, and the degree of disability of the stroke.^[19]^ The quality of sexual life of patients after IS has decreased, which seriously affects the patient's life and family relationship. During the follow-up, if there was a significant decline in sexual function or psychological problems, the patients would get rational treatment.

It can be seen from the results of MRI that obstruction of the frontal and lateral ventricles of the brain may have an effect on erectile function. The structure of each lateral ventricle is similar to a C-shape, starting at a lower-angled lobes, the body traveling in the parietal and frontal lobes, and ending most in the interventricular space, where each lateral ventricle is connected to the central third ventricle. Along the path, a posterior angle extends rearward into the occipital lobe, extending further into the frontal lobes with an anterior horn. Studies have shown that the cortical area that promotes erectile function during sexual stimulation is located in the medial frontal and lower frontal lobes, both of which are areas of the lateral ventricle. Ischemic injury in these areas may affect erectile function.^[20]^ Similar studies have confirmed that post-stroke erectile function decline is associated with lesions in the right occipital-parietal region, with visual and somatosensory information, as well as lesions in the left and adjacent sacral regions, with the primary role of generating and mapping visceral arousal states.^[21]^

This study also investigated the psychological status of patients after onset, and proved that patients in the ED group had more serious anxiety and depression. The meta-analysis showed that the proportion of patients with anxiety after stroke was significantly higher, and the prevalence of post-stroke depression in the general population was about 29%.^[22]^ At the same time, depression can also lead to a variety of male diseases, especially the occurrence of ED. Almost all men with depression have different degrees of ED,^[23]^ affecting male sexual desire, penile erection, ejaculation function, and other aspects. In patients with depression, platelet nitric oxide synthase activity decreases, plasma NO concentration decreases.^[24]^ In addition, patients have an emotional state of anxiety for a long time, leading to sympathetic excitation, promoting the release of norepinephrine, causing vasoconstriction, and decreasing plasma NO concentration. In the central nervous system, stroke patients have hypothalamic-pituitary-gonadal axis function decline, due to reduced gonadotropin secretion, resulting in less androgen, loss of libido, which leads to ED. Therefore, the presence of ED after stroke may be the result of anxiety and depression.^[25,26]^

Previous studies have shown that cardiovascular risk factors such as diabetes, high blood pressure, smoking, and hyperlipidemia also have an impact on ED. Because they promote changes in arteriosclerosis and subsequent blood supply to endothelial dysfunction.^[27]^ In this study, we confirmed that stroke and ED have common risk factors, including hypertension and hyperlipidemia. The diameter of the penile blood vessels is smaller than that of large blood vessels such as cardiovascular and cerebrovascular vessels, and is more susceptible to factors such as arteriosclerosis and inflammatory factors. Therefore, penile blood vessels are more susceptible to first-ever effects such as hypertension and hyperlipidemia than cardiovascular diseases. Multivariate logistic regression analysis demonstrated that taking antihypertensive drugs was an independent risk factor for stroke ED. Studies have shown that antihypertensive drugs can lower blood pressure, induce systemic vascular remodeling, and cause arteriosclerosis, causing narrowing of the cavernous lumen of the cavernous body, and reducing blood flow to the penile artery, which is insufficient to produce or maintain an erection.^[28]^ At the same time, hypertension can cause ED by damaging vascular endothelial cells, causing endothelial dysfunction and structural abnormalities, weakened endothelium-dependent diastolic function, or enhanced endothelium-dependent contractile function. In the study, more than half of IS patients used antihypertensive drugs (67.9%).^[29]^

In this article, there were still some limitations. For example, as the sample size of the study was relatively insufficient, and there was a lack of investigation on the psychological status on patients’ spouses. As this study paid more attention to recovery of sexual function of patients 6 months post IS. So in the future, investigation will be designed to evaluate the neurological status and ED symptoms in different time points and care about the association between IS severity with the ED.

## 5 Conclusions

The results of this study indicated that the incidence of ED was higher in male stroke patients. Antihypertensive drug, depression and anxiety were the independent risk factors for ED in male stroke patients. Paying attention to diagnosis and treatment of ED after male stroke has certain significance for improving their quality of life.

## Author contributions

**Conceptualization:** Hengheng Dai, Jisheng Wang, Bin Wang.

**Data curation:** Qi Zhao.

**Project administration:** Jianxiong Ma, Haisong Li.

**Resources:** Xihao Gong.

**Software:** Lu Wang, Binghao Bao.
